# Rheological Behavior of Clay Tailings in the Presence of Divalent Cations and Sodium Polyacrylate: Insights from Molecular Dynamics Simulations

**DOI:** 10.3390/polym16213091

**Published:** 2024-10-31

**Authors:** Jahir J. Ramos, Steven Nieto, Gonzalo R. Quezada, Williams Leiva, Pedro Robles, Fernando Betancourt, Ricardo I. Jeldres

**Affiliations:** 1 Departamento de Ingeniería Química y Procesos de Minerales, Facultad de Ingeniería, Universidad de Antofagasta, Antofagasta 1240000, Chile; jahir.ramos.ibanez@ua.cl; 2 Advanced Mining Technology Center (AMTC), Universidad de Antofagasta, Antofagasta 1240000, Chile; yeison.nieto.mejia@ua.cl; 3 Escuela de Ingeniería Civil Química, Facultad de Ingeniería, Universidad del Bio-Bio, Concepción 4030000, Chile; grquezada@ubiobio.cl; 4 Facultad de Ingeniería, Arquitectura y Diseño, Universidad San Sebastián, Sede Concepción, Concepción 4030000, Chile; williams.leiva@uss.cl; 5 Escuela de Ingeniería Química, Pontificia Universidad Católica de Valparaíso, Valparaíso 2340000, Chile; pedro.robles@pucv.cl; 6 Department of Metallurgical Engineering, University of Concepción, Concepción 4030000, Chile; fbetancourt@udec.cl

**Keywords:** rheological behavior, clay tailings, divalent cations, sodium polyacrylate, molecular dynamics

## Abstract

This study analyzes the behavior of sodium polyacrylate (NaPA) as a rheological modifier for clay-based tailings. Special emphasis is placed on the impact of calcium and magnesium ions in industrial water, which are analyzed through rheograms, zeta potential measurements, and molecular dynamics simulations. The results are interpreted as electrostatic interactions, steric phenomena, and cation solvation. This interpretation integrates experimental studies with microscopic analyses, employing molecular dynamics simulations to elucidate the underlying mechanisms. In all cases, a decrease in the yield stress of synthetic slurries is observed as the dosing of NaPA increases due to greater repulsion between tailings particles through an increase in electrostatic repulsion and larger steric forces that hinder agglomeration. However, efficiency is reduced in the presence of divalent cations as zeta potential measurements suggest a reduction in the electrical charges of the particles and the polymer, making its application more challenging. The differences obtained in the presence of calcium compared to magnesium are explained in terms of the solvation of these ions and their impact on the polymer conformation in solution and adsorption on the mineral surfaces. This explanation is reinforced by molecular dynamics studies, which indicate that polymer adsorption on minerals depends on the type of mineral and type of ion. Particularly for quartz, the highest adsorption of NaPA occurs in the presence of calcium, whereas for a kaolinite surface, the highest polymer adsorption is obtained in the presence of magnesium. The competitive effect of these phenomena leads to the rheological behavior of the tailings being dominated by the effects originating in the clay.

## 1. Introduction

Water scarcity represents a significant challenge for the sustainability of the mining industry, given that water is an essential resource in almost all operations of a concentrator plant, including comminution, classification, flotation, and tailings management, which are essential processes. In this context, tailings thickening plays a critical role in water recovery. This process occurs in large equipment known as thickeners, where the flotation tailings concentrate through sedimentation, resulting in two output streams: (a) an overflow of clarified water for recovery and reuse in upstream operations and (b) an underflow of thickened pulp removed from the thickener bottom and sent to the tailings storage facilities. 

The flowability of pulps and their transport strategies are largely determined by rheological properties such as yield stress and viscosity. Therefore, it is crucial to exercise caution when reaching high solids concentration values in the underflow, considering its impact on rheological parameters. Depending on the geographical context of the plant, it might not be feasible to transport highly thickened slurries (e.g., paste tailings), which would limit the water recovery potential. Clay minerals, which are present in considerable amounts in copper and copper-gold deposits, are characterized by laminar particle morphology, fine-sized particles, and anisotropic character; the rheological properties of their suspensions become increasingly complex compared to spherical morphology [1,2]. In general, the presence of clay minerals affects the valuable mineral processing circuit, finding inefficiencies in the stages of concentration, dewatering, pulp transport, and tailings deposition [3].

To mitigate this limitation, methods that improve tailings fluidity can be employed, such as applying shear thinning strategies or using rheological modifying reagents suitable for challenging environments such as highly clayey minerals or low-quality waters. For example, Robles et al. [4] demonstrated that seawater produces greater particle agglomeration in a concentrated kaolin suspension than distilled water. Seawater contains counterions such as Na^+^ and Mg^2+^, forming cationic bridges between the anionic kaolin particles, causing their aggregation and higher rheological properties. Similarly, Reyes et al. [5] studied the effect of the seawater/process water ratio on the rheological properties of magnetite tailings. The results indicated that the yield stress is highly sensitive to salinity, while the plastic viscosity showed a comparatively weaker dependence on this variable.

Conversely, Jeldres et al. [6] discovered that in kaolin pulps flocculated with anionic polyacrylamide, the yield stress decreases with increasing salinity (study performed at pH 5.5). This is due to the weakening of the face-edge bonds of the clay mineral under the influence of salinity. In a separate study, Jeldres et al. [7] examined the effect of cation size on the rheological properties of silica suspensions. Their findings revealed that larger cations, such as cesium and potassium, tend to adhere to the silica surface more than smaller cations, such as sodium and lithium. This increases the number of ionic bonds that unite the particles, resulting in higher rheological parameters such as yield stress, complex viscosity, and viscoelastic moduli. These results suggest that ‘breaker’ ions strengthen the interactions between silica particles, while ‘maker’ ions have the opposite effect.

The current research explores the intricate interaction between various cations and rheological modifying reagents. These reagents, including some inorganic ones [8], play a crucial role in altering the surface charge of mineral particles, thereby regulating the electrostatic attraction/repulsion between them. For instance, the work of Leiva et al. [9] is a testament to this, as they successfully modified the rheological properties of clayey tailings using sodium pyrophosphate, a tetravalent anionic molecule. This modification increased the electrostatic repulsion of the particles, even in a saline medium, leading to a substantial reduction in the yield strength of the suspension.

On the other hand, organic additives act through a combination of electrostatic and steric forces; the latter is related to the molecular weight of these reagents [10]. For example, Shakeel et al. [11] investigated the rheological properties of kaolin suspensions after adding a series of biopolymers (xanthan gum (XG), sodium carboxymethylcellulose (CMC), potato starch (PS), chitosan (Ch), and apple fiber (AF)) by varying the amount of clay and the type of biopolymer. The rheological results showed that adding different biopolymers to the kaolin suspensions significantly influenced the viscosity and the viscoelastic moduli. In the case of chitosan, a strong reduction in viscosity was observed compared to the pure kaolin suspension, which can be attributed to the charge neutralization phenomenon.

Moreover, the role of sodium polyacrylate in stabilizing kaolin pulps in seawater is a significant finding. Robles et al. [4] conducted a thorough analysis of this, and their results indicated that the rheological properties of the pulps were significantly reduced after adding the polymer. Subsequently, Jeldres et al. [12] furthered this research by studying the stabilization of synthetic tailings rich in clays such as quartz-kaolin and quartz-montmorillonite in seawater at pH = 8. Their findings revealed that the yield strength of both tailings decayed exponentially with increasing dosage of sodium polyacrylate. This was accompanied by a slight increase in the magnitude of the negative zeta potential and a clear shift of the particle size distribution toward finer sizes. The proposed stabilization mechanism involves steric repulsion, where the long and flexible chain structure of polyacrylate wraps and surrounds solid particles, preventing them from agglomerating and settling.

In the search for methodologies that allow improving the understanding of the interaction between salts, surfaces, and reagents, molecular dynamics has emerged as a great strategy to analyze properties such as adsorption density, quantify types of bonds, and determine the conformation of the molecule in solution, among other phenomena that influence the efficiency of the rheological behavior of a suspension. Some studies have described the behavior of different polymers on quartz and kaolinite surfaces, including evaluating the interaction and behavior of monovalent ions in the adsorption of sodium polyacrylate on quartz surfaces. Quezada et al. [13] observed that the formation of cationic bridges was more pronounced with brines of small cations (maker structure) such as Li and Na, and these bridges contribute to the formation of hydrogen bonds and hydrophobic bridges, enhancing the adsorption stability in these cations. In contrast, interactions are weaker in the case of K and Cs, larger cations with a breaker structure. Significant scientific progress has been made in understanding the mechanisms of colloidal and molecular interaction between rheological modifiers and the surfaces of particles in mining tailings, such as quartz or various clays. The literature shows a series of analyses of sodium polyacrylate in the rheological response of suspensions composed of various qualities of water, that is, of different types of ions present. There is interest in exploring the performance of sodium polyacrylate in low-quality waters, which typically have high calcium and magnesium content. These divalent cations have notable impacts on flotation operations [14], thickening [15], and rheology [16]; however, to date, none of the literature reveals their consequences on the performance of a rheological modifier, which is the focus of the present study. Therefore, rheological analysis, zeta potential, and molecular dynamics are conducted to enhance the current understanding of the behavior of sodium polyacrylate in low-quality waters, commonly found in mineral processing, with specific attention to regions experiencing water scarcity. The article is organized as follows; Section 2 is devoted to the methodology, which will describe the experimental materials, setup, and procedure, as well as the framework for the molecular dynamic simulation. Section 3 shows the results and the discussion. The article ends with a conclusion section where the main findings of the research are summarized. This study seeks to understand the impact on the rheological response of the addition of sodium polyacrylate in pulps dispersed in waters with high concentrations of Ca^2+^ and Mg^2+^, two important cations present in seawater, an alternative to water scarcity in the mining industry. The emphasis on these cations is due to their divalent nature, which has greater agglomerating power of the solid particles of the tailings compared to the monovalent cations (Na^+^, K^+^) present in seawater, resulting in higher values of the rheological properties of the pulps. The addition of sodium polyacrylate seeks to mitigate this effect.

## 2. Materials and Methods

### 2.1. Materials

**Figure 1:**
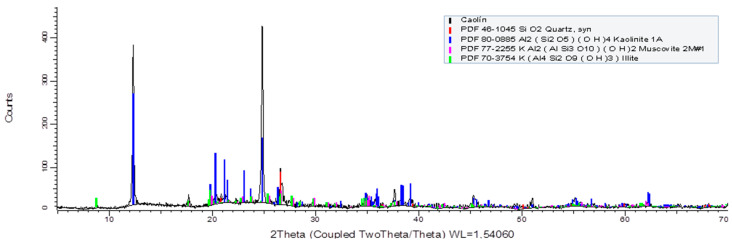
X-ray diffraction pattern of kaolin.

Kaolin particles from Ward’s Science (Clay Spur, WY, USA) were used. The mineralogical composition was determined using a Bruker X-ray diffractometer (Bruker, Billerica, MA, USA), Model D8 advance. Quantitative analysis processed with TOPAS (Total Pattern Analysis Software, Siemens S.A., Las Condes, Chile) indicated that the sample contained 65.3% by weight of kaolinite (Al_2_ (Si_2_O_5_) (OH)_4_), 20.6% of illite (K(Al_4_ Si_2_O_9_(OH)_3_), 11.9% of quartz (SiO_2_), and 2.2% of muscovite ((H,K)AlSiO_4_. The diffractogram of kaolin is presented in Figure 1. Quartz particles (SiO_2_) were obtained from Donde Capo (Santiago, Chile), and the sample was crushed and sieved to obtain 100% of the sample under mesh #270 (ASTM E 11). XRD analysis shows that it contains 100% SiO_2_.

Sweeps were carried out with different concentrations of MgCl_2_*H_2_O (0.1 M–1 M) and CaCl_2_ (0.1 M–1 M).

**Figure 2:**
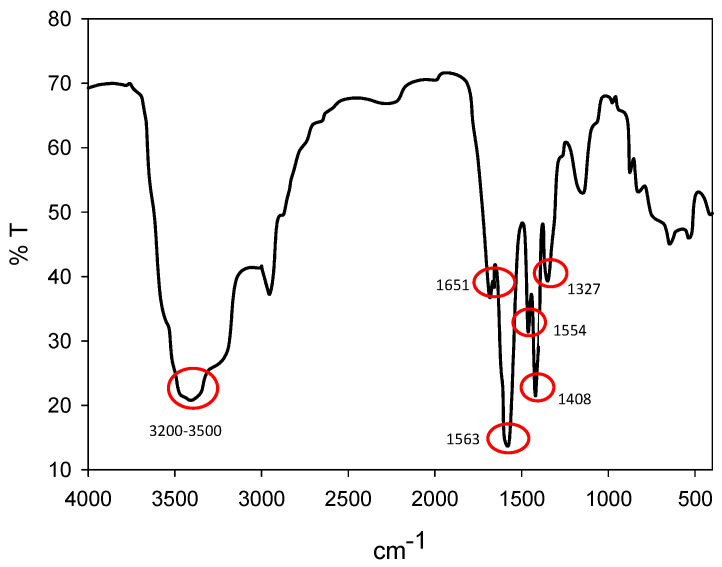
FTIR of sodium polyacrylate (NaPA).

Sodium polyacrylate with a molecular weight of 5100 g/mol was obtained from Sigma-Aldrich. A brand Fourier infrared spectrometer model FT/IR-4600 (JASCO Corporation, Tokyo, Japan) was used for the infrared measurement of the reagent. In Figure 2, the stretching vibrations of the hydroxyl group can be seen between the wavelengths of 3200–3500 cm^−1^. At wave number 1651 cm^−1^, absorption bands corresponding to the C-OH deformation vibrations can be observed. The asymmetric and symmetric stretching vibrations of the carboxyl anions -COO- between the waves 1408 and 1563 cm^−1^ is typical of carboxylic acid salts; finally, the oscillation (1327 cm^−1^) and deformation (1454 cm^−1^) characteristic of CH_2_ are observed.

### 2.2. Zeta Potential

Zeta potential measurements were carried out on 1 wt% quartz and kaolin suspensions at pH = 8. Before the measurements, the suspensions were stirred for 10 min, after which sodium polyacrylate was added at a concentration of 2000 g/t. Measurements were performed using an Anton Paar Litesizer (Graz, Austria) employing the electrophoretic light scattering technique. Data analysis was performed using Kalliope software (version 3.8.2) with the Smoluchowski model and a Henry factor 1.5. Samples were analyzed in the Omega model cuvette. Results were compared with control measurements conducted without the reagent. The solutions utilized in the experiments are elaborated upon in Section 2.1.

### 2.3. Rheological Measurements

Synthetic tailings with a quartz-kaolin composition and a solids concentration of 60 wt% at pH = 8 were prepared, where 70% of the solid corresponds to quartz and 30% to kaolin. The mixture was stirred for one hour at 600 rpm to ensure complete homogenization of the pulp. Subsequently, the pH was adjusted to 8 by the addition of NaOH. After reaching the desired pH, sodium polyacrylate (NaPA) was added and stirring continued for 15 min. A 22 mL aliquot was removed and placed in the measuring cup model “CC27-XL-SS” using the conical sensor “CC7/S 83307” (Anton Paar Company, Graz, Austria). Rheological measurements were performed using an Anton Paar MCR 102 rheometer and the Rheocompass software (version 1.3) for the rheological characterization of the sample.

Flow curves were created and fitted to the Herschel–Bulkley model. The measurement was conducted on a logarithmic time ramp with an initial duration of 20 s and a final duration of 1 s. Concerning the shear rate, it started at 20 s^−1^, increasing linearly to the shear stress value of 500 s^−1^. The sample temperature remained constant at 25 °C. The rheological parameter (yield stress) was determined by fitting the experimental data to the constitutive equation of the Herschel–Bulkley model (refer to Equation (1)):

(1) $$\tau \;={\;\tau }_{0}+{\;\mathrm{kY}}^{n}$$

where τ is the shear stress, Y the shear rate, $\tau_{0}$ is the yield stress, k the consistency index and n the flow index. The Herschel–Bulkley model is the one that best fits the rheological behavior of mining pulps. Generally, mining pulps have pseudoplastic behavior, that is, their viscosity decreases with shear rate. If we add to this behavior the presence of a yield stress (which could occur in this study) we obtain a Bingham pseudoplastic that can be modeled by the Herschel–Bulkley equation. The solutions used are those named in Section 2.1.

### 2.4. Computer Simulations

As part of our analysis, we conducted molecular dynamics simulations applying Newton’s equations to energy-conservative potentials. Our thorough methodology was designed to closely examine the affinity of the NaPA polymer to quartz and kaolinite surfaces. The simulation system was based on the methodology described by Quezada et al. [13], which introduces a quartz or kaolinite crystalline surface into a simulation box immersed in an aqueous solution containing dissolved NaPA and CaCl_2_ or MgCl_2_ salts.

The CLAYFF [17] energy potential modeled the mineral surfaces, GAFF [18] for NaPA, and parameters proposed by Li et al. [19] for divalent ions. These parameters established a strong foundation for our simulations, lasting 100 ns with time steps of 2 fs. The simulations were conducted under a canonical ensemble at a constant volume and temperature (NVT) [20,21] using the Gromacs (version 2021.2) software [22]. To analyze the adsorption frequency of NaPA and the resulting adsorption configurations, we used the tool gmx mindist to analyze the minimum distance of the NaPA to the surface. We also used gmx gyrate to measure the radius of gyration of the adsorbed NaPA and gmx density to calculate the charge profile above the surface. The simulation conditions are summarized in Table 1.

## 3. Results and Discussion

This section of the study is structured in three main parts. Initially, a rheological analysis of synthetic tailings composed of quartz and kaolin mixtures is carried out. This analysis aims to evaluate the impact of sodium polyacrylate on different calcium chloride and magnesium chloride solutions. In the second part, the rheological results are correlated with electrostatic changes on the particle surfaces using zeta potential measurements. This facilitates the interpretation of particle dispersion mechanisms in solutions. Finally, the third section uses molecular dynamics simulations to address changes in the polymer conformation and its interaction with mineral surfaces. This approach allows a deeper understanding of the polymer adsorption mechanisms, providing critical insights for the practical application of these findings.

### 3.1. Rheological Analysis

#### 3.1.1. Effect of Sodium Polyacrylate (NaPA)

**Figure 3:**
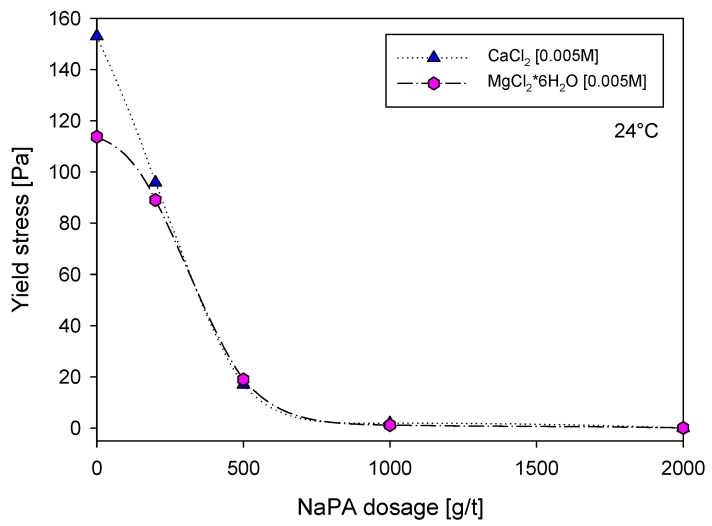
Yield stress of quartz-kaolin pulps as a function of NaPA dosage for liquid phase qualities composed of 0.005 M MgCl_2_*6H_2_O and 0.005 M CaCl_2_.

Figure 3 illustrates the impact of dispersant dosage on the yield stress of kaolin suspensions prepared in calcium chloride and magnesium chloride brines, both at a concentration of 0.005 M and pH = 8. Under these conditions, quartz-kaolin particles have a negative surface charge, which favors the adsorption of cations. The results show that, without a dispersant, the yield stress reaches maximum values of 153 Pa for calcium and 113 Pa for magnesium. This suggests that calcium ions have a greater adsorption capacity on quartz-kaolin particles, promoting more significant aggregation and increased yield stress. This highlights the enlightening role of cation adsorption in the aggregation of quartz-kaolin particles.

Adding the NaPA dispersant resulted in a significant reduction in yield stress, showing that the pulp acquires non-viscoelastic properties at a 1000 g/t dosage for both types of ions. Initial differences in yield stresses between the ions were observed, attributed to a more robust hydration shell in magnesium than calcium, resulting in lower yield stress values for magnesium. However, these differences are significantly reduced with doses higher than 250 g/t NaPA, to the point of being almost imperceptible between both systems. This reduction in yield stress can be attributed to the NaPA dispersant’s ability to disrupt the aggregation of quartz-kaolin particles, thereby reducing the overall yield stress.

#### 3.1.2. Effect of Ca^2+^ and Mg^2+^

**Figure 4:**
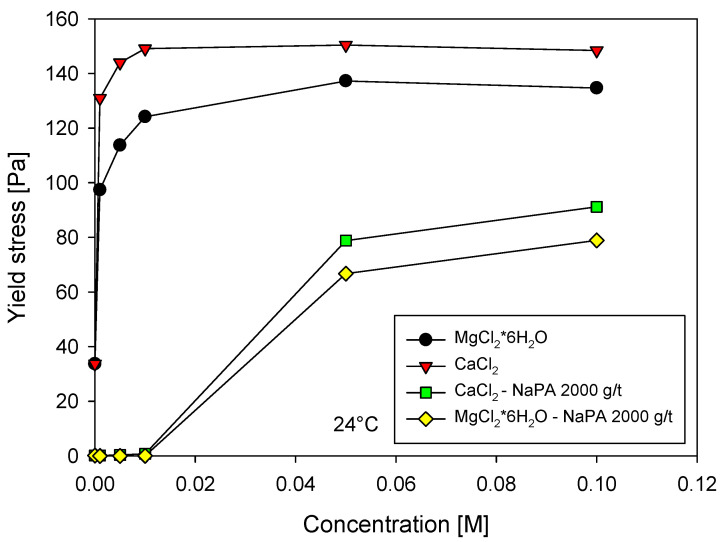
Yield stress as a function of the molar concentration of Ca^2+^ and Mg^2+^ in the absence and presence of NaPA.

Our study on quartz-kaolin pulps at pH = 8, with varying CaCl_2_ and MgCl_2_*6H_2_O concentrations from 0 M to 0.1 M and NaPA doses of 0 and 2000 g/t, as depicted in Figure 4, revealed that the yield stress increases with the ion concentration until it reaches a steady state in both cases. This increase is due to the negatively charged quartz-kaolin particles attracting divalent ions, which facilitate coagulation, increasing pulp stiffness and interfering with its fluidity.

The data in Figure 4 confirm that, in the absence of NaPA, the yield stress increases rapidly, reaching maximum values of 130 Pa for magnesium and 150 Pa for calcium. The intriguing stabilization of the yield stress from a salt concentration of 0.03 M suggests a saturation of ions in the particles’ electrical double layer, which has significant implications for our understanding of pulp behavior under varying ion concentrations.

The inclusion of NaPA at a high dose results in a significant reduction in the yield stress for low salt concentrations, reaching practically zero values. However, above 0.01 M salt concentration, the yield stress increases again, indicating that the ions can be adsorbed together with NaPA. Furthermore, the greater increase in yield stress with calcium compared to magnesium reflects the greater capacity of calcium to adsorb on minerals, which can inspire new approaches to mineral processing.

To further elaborate on the differences observed between magnesium and calcium cations, Table 2 details the various chemical properties of these cations. The ionic radius, determined by the loss of electrons from the outer shell, influences the other properties reported in the table. Because magnesium has a smaller radius than calcium but maintains the same electrical charge, it results in a higher electrical surface density. This translates into higher hydration energy and entropy, leading to more significant adsorption of water molecules and a higher coordination number [23]. These properties indicate that magnesium ions experience difficulty in adsorbing directly onto minerals due to their robust hydration shell. In contrast, calcium, which can rearrange water molecules in its environment more efficiently, adsorbs more effectively onto mineral surfaces, promoting particle aggregation and increasing yield strength.

### 3.2. Zeta Potential

**Figure 5:**
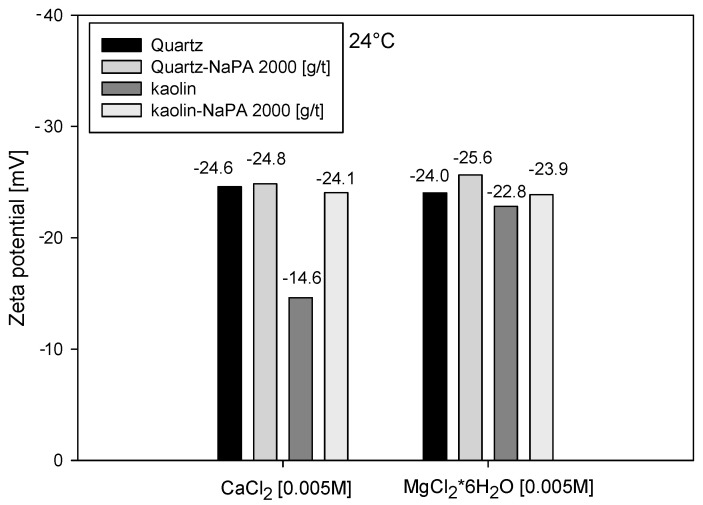
Zeta potential of quartz and kaolin in the absence and presence of NaPA (2000 g/t) for water qualities composed of 0.005 M MgCl_2_*6H_2_O and 0.005 M CaCl_2_ solutions. All at pH = 8.

Zeta potential measurements were conducted to assess the role of electrostatic forces in quartz and kaolin minerals’ aggregation and dispersion processes, which were studied separately. The measurements were conducted in 0.005 M MgCl_2_*6H_2_O and 0.005 M CaCl_2_ solutions under controlled conditions in the absence and presence of sodium polyacrylate (NaPA) at pH = 8, as shown in Figure 5. In cases without NaPA, the zeta potentials for quartz resembled those of calcium and magnesium. However, in kaolin, a more pronounced effect on the zeta potential was observed in the presence of calcium, which can be attributed to its higher negative charge density favoring the adsorption of calcium on magnesium, as detailed in Table 2. The addition of NaPA significantly altered the zeta potential in all cases, a clear indication of its adsorption on mineral surfaces. Chemically, NaPA is an anionic polymer (COO—) that increases the particles’ negative charge when adsorbed on mineral surfaces, as reflected in the observed changes in zeta potentials.

**Figure 6:**
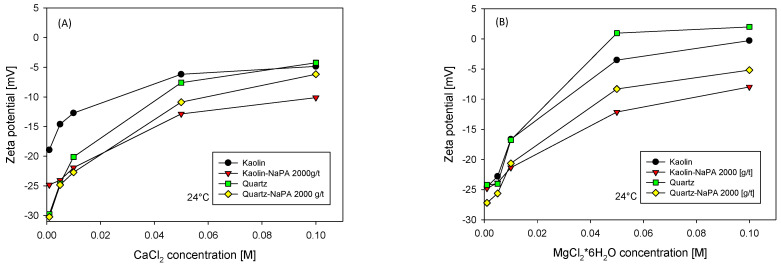
Zeta potential of quartz and kaolin in solutions of different salinities in the absence and presence of sodium polyacrylate at pH = 8: (**A**) CaCl_2_ and (**B**) MgCl_2_*6H_2_O.

Expanding on the results observed in Figure 5, a study was performed by increasing the concentration of the MgCl_2_*6H_2_O and CaCl_2_ salts, evaluating the zeta potential in the absence and presence of NaPA (2000 g/t), as shown in Figure 6. The observed trends indicate an apparent neutralizing effect of the ions on the mineral surfaces, manifested by a decrease in the absolute value of the zeta potential values. The impact of NaPA on the measurements is moderate at low salt concentrations but intensifies as the salt concentration increases, leading the zeta potential toward more negative values [25]. These findings are consistent with the yield stress results presented in Figure 4, where the yield stress increases at lower zeta potential values. Furthermore, it is noted that in the case of quartz with magnesium, the zeta potential is neutralized more than with calcium, probably because magnesium ions influence the diffuse layer more, thus generating a zeta potential that tends to be positive.

### 3.3. Simulation of NaPA Adsorption on Minerals

**Figure 7:**
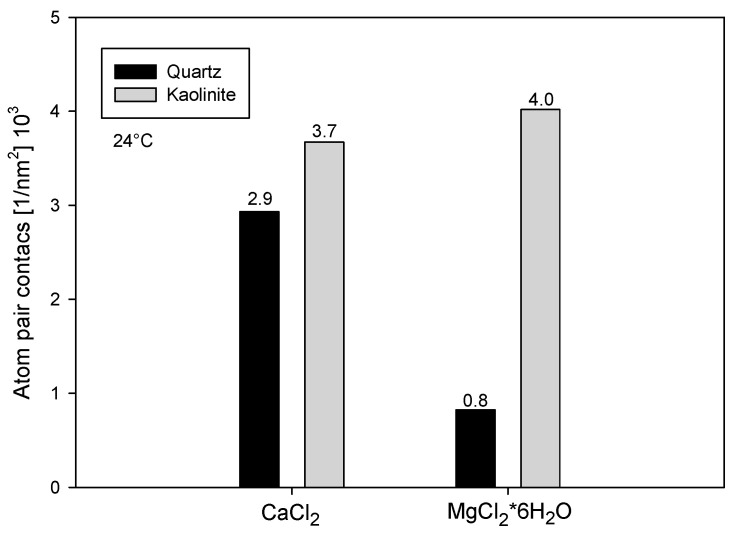
Averaged adsorption of NaPA on the surfaces in the presence of salts. Ka: kaolinite, Qz: quartz, Mg: magnesium chloride, Ca: calcium chloride.

As a complement to the experimental results, molecular dynamics simulations were conducted to quantify the adsorption of the NaPA polymer on quartz and kaolinite surfaces in waters with calcium and magnesium chloride. These simulations provide atomic-level insights into the interactions that are difficult to measure experimentally. Figure 7 illustrates the affinity frequency of the polymer with the surfaces, indicated by the number of contacts between the surface atoms and the polymer per unit of surface area. This parameter is crucial for understanding how well the polymer adsorbs onto the mineral surfaces. In systems containing MgCl_2_*6H_2_O, the affinity of NaPA is higher on kaolinite (0.004 nm^−2^) than on quartz (0.001 nm^−2^), suggesting stronger interactions with kaolinite in the presence of magnesium ions. In systems with CaCl_2_, the affinity is also greater on kaolinite (0.004 nm^−2^) than on quartz (0.002 nm^−2^).

The higher charge density of kaolinite can explain this phenomenon compared to quartz, which facilitates a greater attraction of cations and, consequently, better adsorption of NaPA on kaolinite.

The higher charge density of kaolinite is likely responsible for this stronger interaction, as it facilitates the attraction of more cations (Ca^2^⁺ and Mg^2^⁺), enhancing the adsorption of the negatively charged NaPA polymer. In contrast, quartz, with a lower surface charge density, attracts fewer cations, leading to weaker polymer adsorption. The difference between calcium and magnesium adsorption is also noteworthy: calcium tends to interact more strongly with quartz, likely due to its ability to shed its hydration shell and bond directly with the mineral surface. Magnesium, on the other hand, typically retains its hydration layer, which reduces its direct interaction with quartz. On kaolinite, however, the adsorption of NaPA is similar for both cations, likely due to kaolinite’s high charge density, which compensates for magnesium’s retained hydration layer by attracting more ions overall.

These findings may explain the observations in Figure 3 and Figure 5, where calcium reduces the yield stress of the quartz-kaolinite mixture more significantly than magnesium. The stronger interaction between calcium and quartz, in combination with its adsorption on kaolinite, likely leads to more efficient dispersion of particles, reducing yield stress. Magnesium’s weaker interaction with quartz may result in less significant yield stress reduction, consistent with its lower affinity for quartz in the simulation results. These combined results highlight the role of mineral surface characteristics and ion-specific interactions in influencing polymer adsorption and the rheological properties of suspensions.

**Figure 8:**
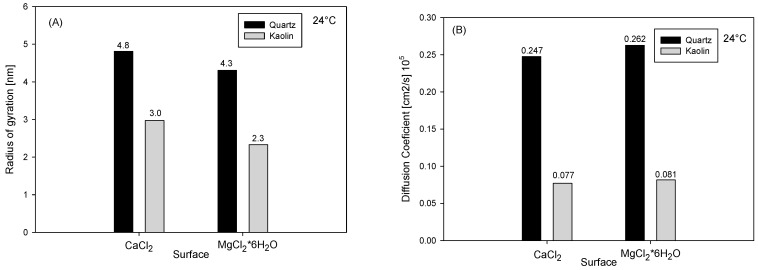
Dynamic characteristics of NaPA in saline solution on mineral surfaces; (**A**) radius of gyration; (**B**) diffusion coefficient.

Characteristics of the adsorbed NaPA were determined by analyzing the radius of gyration to assess the degree of coiling (Figure 8A) and the diffusion coefficient to measure its mobility during the simulation (Figure 8B). The radius of gyration results reveal that NaPA adopts more extended structures in the vicinity of quartz compared to kaolinite. This behavior is likely due to the higher charge density of kaolinite, facilitating a higher concentration of ions interacting with NaPA. Consequently, there is a higher degree of coiling near kaolinite, which further affects its ability to disperse.

In solutions containing calcium, the degree of coiling of NaPA is lower than in those containing magnesium. Although magnesium has a lower adsorption capacity, its presence in the aqueous solution creates steric hindrances contributing to higher polymer coiling. Furthermore, the mean square displacement of the molecules was analyzed to determine the diffusion coefficient of NaPA when it is not adsorbed (Figure 8B). The results indicate that diffusion is faster in the presence of quartz than in kaolinite. This is because kaolinite has a higher ion concentration in its vicinity, hindering the free diffusion of NaPA. The differences in behavior between magnesium and calcium presence are slight; however, NaPA exhibits greater mobility in the presence of magnesium clusters.

**Figure 9:**
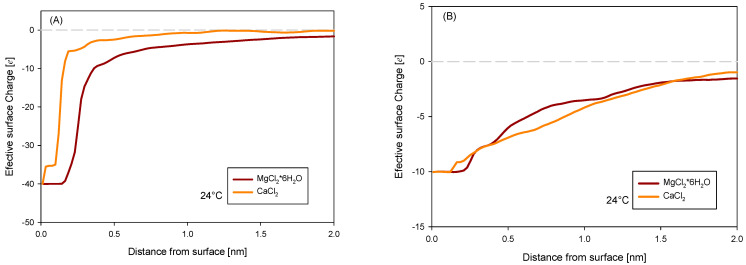
Net charge on (**A**) kaolinite and (**B**) quartz in different salt waters in the presence of NaPA.

Another analysis was the net charge obtained on the surface. In Figure 9A, this profile is graphed from the surface with a charge of −40e for kaolinite. It can be analyzed that in the systems with calcium, the adsorption of ions is greater, allowing the neutralization of the system to a greater extent and helping the polymer to be adsorbed. This stronger interaction between calcium and kaolinite suggests that calcium ions play a critical role in reducing surface charge, which enhances the binding of NaPA. Magnesium, in these cases, decreases this interaction because it is not adsorbed on the surface like calcium, likely due to its retention of a hydration layer, which reduces its direct interaction with the surface.

In the cases with quartz surfaces, the electrical effect is comparatively weaker in both cases with salts. Magnesium slightly neutralizes in a range of 0.5 to 1 nm and performs marginally better than calcium, likely due to the smaller ionic radius of magnesium, which allows it to remain closer to the surface. Even so, it is low compared to what is shown with kaolinite, indicating that quartz’s lower charge density reduces the overall adsorption potential for both cations.

**Figure 10:**
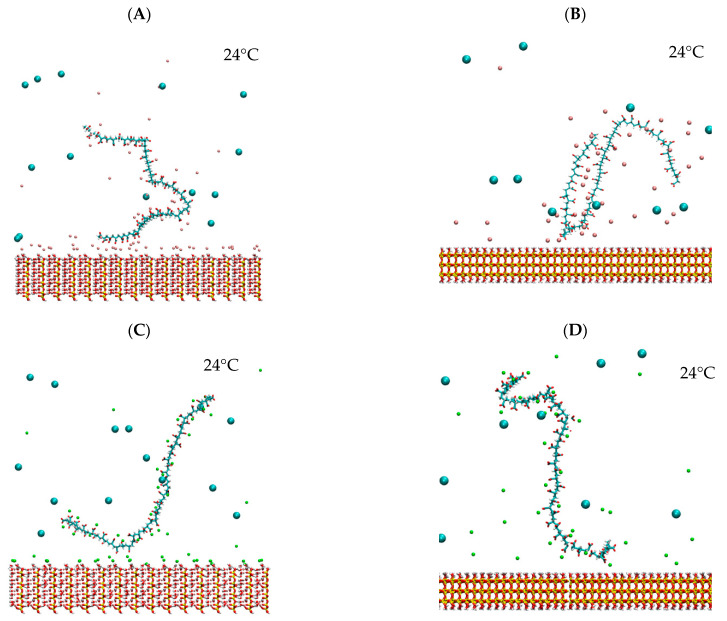
Configurations obtained from the adsorption of NaPA on kaolinite and quartz surfaces (**A**) Kao-linite–MgCl2, (**B**) Quartz–MgCl2 (**C**) Kaolinite–CaCl2, (**D**) Quartz–CaCl2. Chloride ions, cyan spheres; calcium ions, green spheres and magnesium ions, pink spheres.

Figure 10 presents the images obtained by molecular dynamics simulation of the adsorptions in the four systems studied. Since NaPA has a single functional group, the carboxylic group (COO—), interactions with the surface occur mainly through cationic bridges facilitated by cations adsorbed both on the surface and NaPA. The resulting configurations show that NaPA is adsorbed punctually, with large portions of the polymer structure remaining exposed to the surrounding medium. This exposure enhances dispersion, especially when the surface is sufficiently coated with NaPA.

In the magnesium-kaolinite system (Figure 10A), more significant adsorption of cations on the surface is observed, reducing repulsion and allowing better interaction of NaPA with the surface. Conversely, in the quartz-magnesium system (Figure 10B), magnesium ions are preferentially adsorbed on NaPA, causing the polymer to roll up and decreasing its interaction with the surface.

For calcium-containing systems, adsorption on kaolinite (Figure 10C) is more stable because calcium ions are closer to the surface, facilitating the interaction of NaPA. This stronger binding likely contributes to the higher stability of the polymer layer, enhancing the uniform coverage of NaPA on the surface. In the case of quartz with calcium (Figure 10D), a lower interaction of calcium with the surface results in punctual adsorption of NaPA, which could be unstable in the long term due to the weaker binding forces. These observations suggest that the most effective interaction occurs in the kaolinite-calcium system, where multiple NaPA groups interact with the surface, demonstrating remarkable synergy between calcium and the polymer. This highlights the importance of both surface properties and cation type in determining the stability and dispersion behavior of NaPA.

## 4. Conclusions

This study evaluated the aggregation and dispersion mechanisms in synthetic quartz-kaolin pulps in the presence of Ca^2+^ and Mg^2+^ ions at pH = 8 and their interaction with sodium polyacrylate (NaPA). The pulps exhibited non-Newtonian behavior. Specifically, pulps with CaCl_2_ showed a higher yield stress than those containing MgCl_2_. This is attributed to the dynamic hydration characteristics of Ca^2+^, which promote more robust bonds between particles.

Although the addition of NaPA did not significantly alter the electrostatic forces, it enhanced particle dispersion through steric repulsion mechanisms, resulting in a notable decrease in yield stress. With increasing concentrations of Ca^2+^ and Mg^2+^ ions, yield stress was observed until it reached a steady state due to the formation of ionic bridges that increase pulp stiffness. This effect is evident in reducing the particles’ surface charge under the influence of the electrolytes.

Molecular dynamics analysis revealed significant differences in the adsorption of NaPA on quartz and kaolinite surfaces. Higher adsorption was observed in the presence of calcium on quartz, followed by magnesium on kaolinite. These differences arise from the higher charge density in kaolinite, which traps more ions on its surface and consequently reduces the mobility of NaPA.

Incorporating NaPA as an anionic polyelectrolyte is a promising strategy to improve the management of clay-rich tailings in concentrator plants at pH = 8. The thickening process enhances water recovery for recirculation while intensifying the pulp’s rheological properties, potentially increasing the energy consumption related to tailings transport.

In a further study, the effects of NaPA on the recovery of Cu-Mo in the froth flotation process will be analyzed.

## Figures and Tables

**Table 1:** Overview of Simulation Parameters in Molecular Dynamics.

System	Surface Dimension (nm^3^)	Surface Charge at pH = 8 (C/m^2^)	The Concentration of Mg or Ca (mol/L)
Kaolinite-NaPA	10.2 × 11.4 × 2.13	−0.114	0.01
Quartz-NaPA	9.83 × 11.0 × 1.27	−0.030	0.01

**Table 2:** Chemical properties of Ca^2+^ and Mg^2+^ [24].

Ion	Ionic Radio (nm)	Heat of Hydration (kcal/mol)	Hydration Number (mol H_2_O/ion)	Entropy of Hydration (cal/mol·K)	Enthalpy of Hydration (kJ/mol)	Electronegativity	Ionic Potential
Ca^2+^	0.099	−377	8–12	50	−1592	1.00	20.20
Mg^2+^	0.065	−456	12–14	64	−1922	1.31	30.77

## Data Availability

The original contributions presented in the study are included in the article, further inquiries can be directed to the corresponding author.
